# P-495. Assessing Growth and Neurodevelopment in HIV-Exposed Uninfected Children: A Ghanaian Case-Control Study

**DOI:** 10.1093/ofid/ofaf695.710

**Published:** 2026-01-11

**Authors:** Narteki Gyapong-Osei, Roberta Tagoe, Irene A Kumi, Gabrielle Obeng-Koranteng, Thaddeus Ohene Peprah, Emmanuel P Abbeyquaye, Marilyn Marbell-Wilson, Christiana A T M Osei-Yeboah, Anthony Enimil

**Affiliations:** 37 Military Hospital, Accra, Greater Accra, Ghana; 37 Military Hospital, Accra, Greater Accra, Ghana; 37 Military Hospital, Accra, Greater Accra, Ghana; 37 Military Hospital, Accra, Greater Accra, Ghana; 37 Military Hospital, Accra, Greater Accra, Ghana; 37 Military Hospital, Accra, Greater Accra, Ghana; Mission Pediatric Clinic, Accra, Greater Accra, Ghana; COY Pediatrics Clinic, Accra, Greater Accra, Ghana; School of Medicine and Dentistry KNUST, Komfo Anokye Teaching Hospital Kumasi, Kumasi, Ashanti, Ghana

## Abstract

**Background:**

The growing population of HIV-exposed uninfected (HEU) children due to effective PMTCT efforts necessitates understanding their long-term outcomes. This study compared the growth and neurodevelopment of HEU children to their HIV-unexposed, uninfected (HUU) peers in Ghana.

**Table I:**
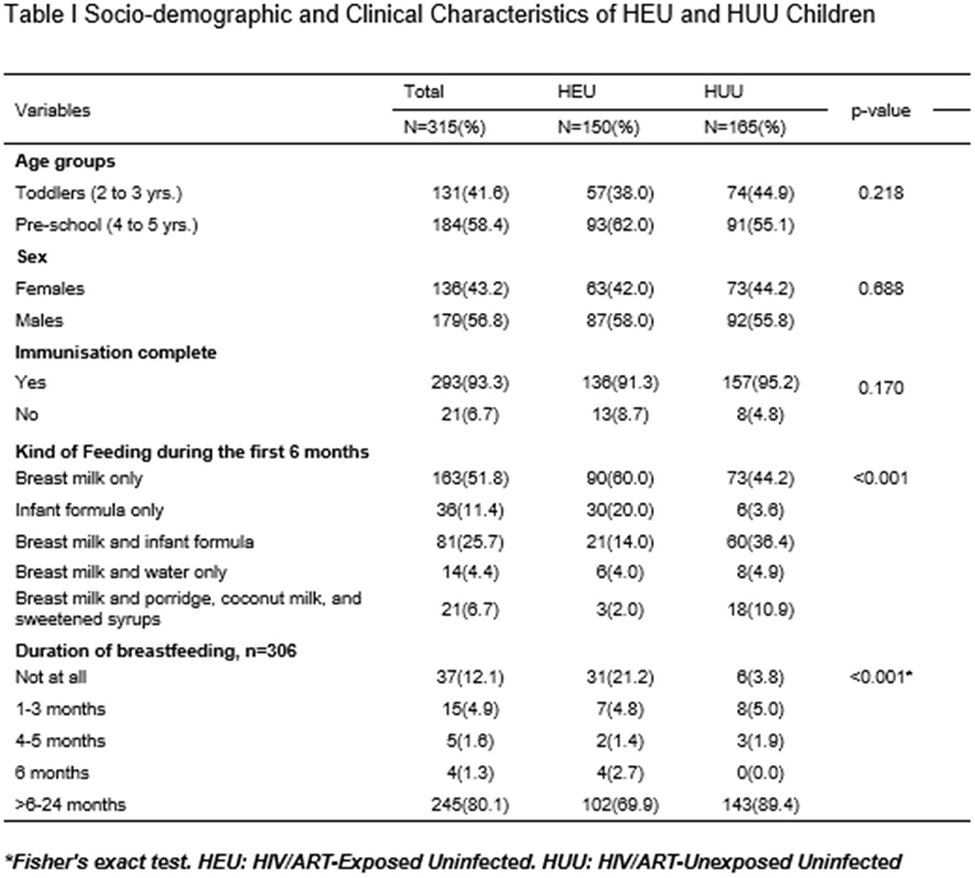
Socio-demographic and Clinical Characteristics of HEU and HUU Children

**Table II:**
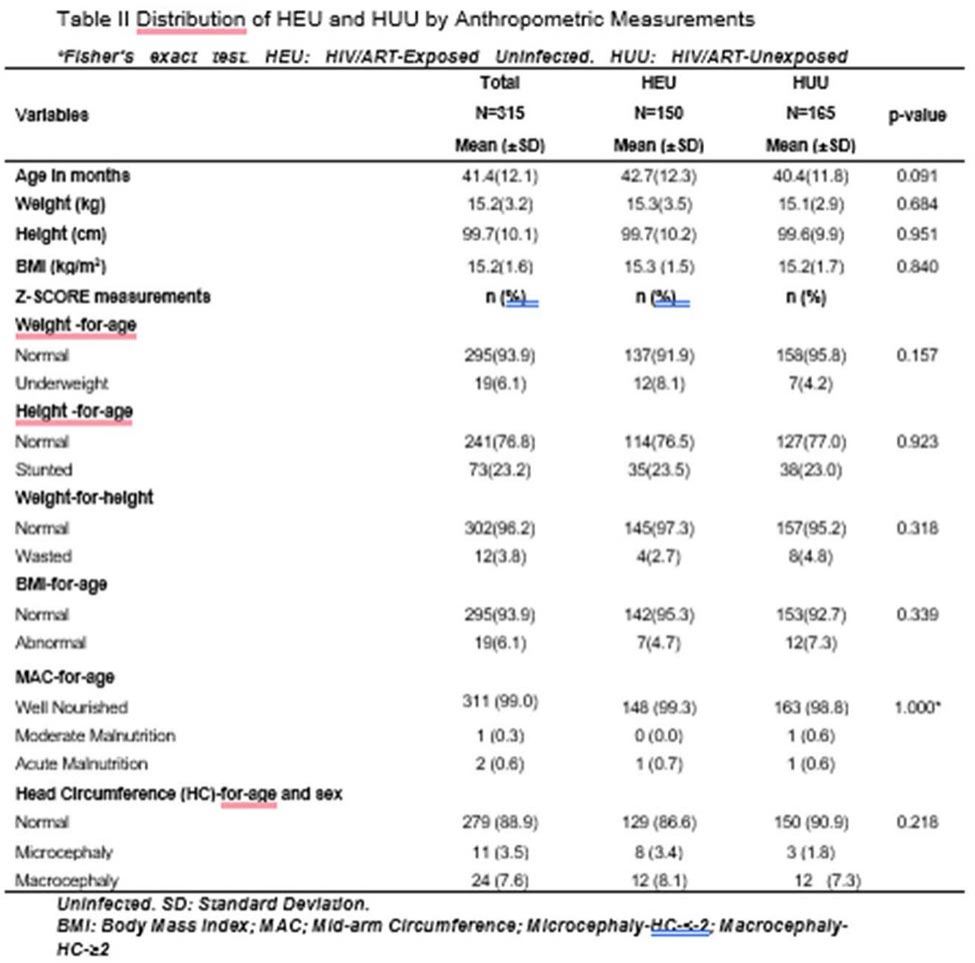
Distribution of HEU and HUU by Anthropometric Measurements

**Methods:**

A hospital-based case-control study was conducted at 37 Military Hospital, Accra, from December 2021 to August 2022. We enrolled 315 children aged 2–5 years—150 HEU and 165 HUU. Maternal/carer sociodemographic data were collected via structured interviews. Nutritional status was assessed through anthropometry, and neurodevelopment was measured using the Malawi Developmental Assessment Tool (MDAT). Bivariate analysis and multivariate logistic regression identified factors associated with adverse outcomes.

**Table III:**
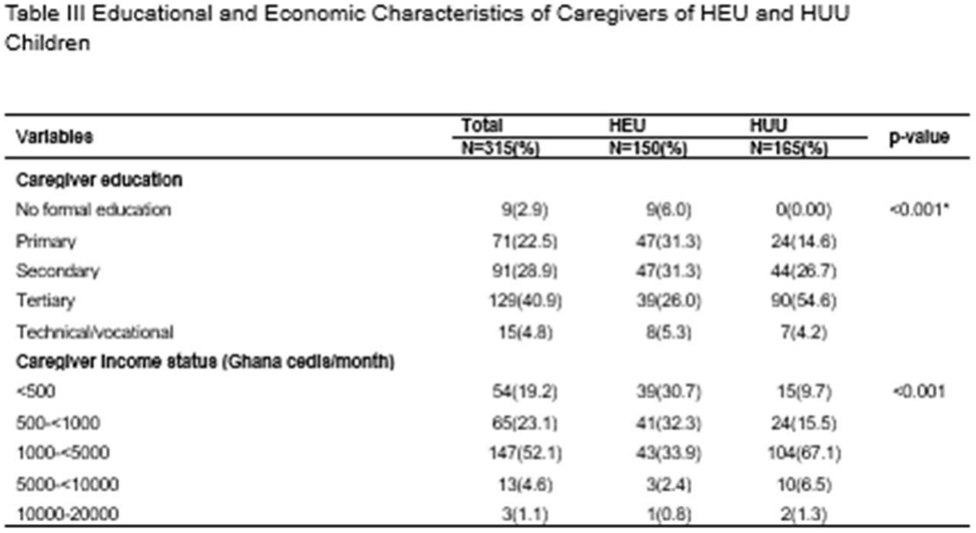
Educational and Economic Characteristics of Caregivers of HEU and HUU Children

**Table IV:**
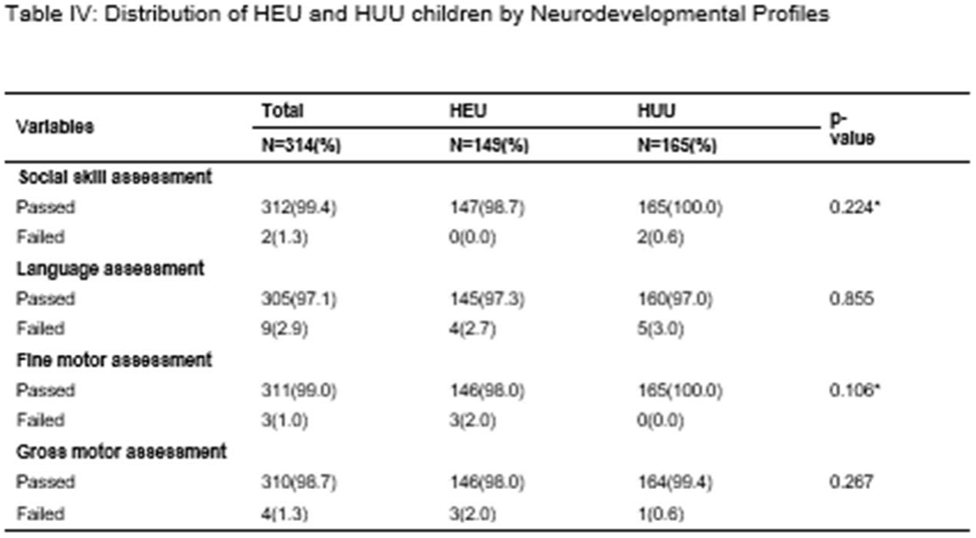
Distribution of HEU and HUU children by Neurodevelopmental Profiles

**Results:**

Normal weight-for-age was observed in 91.7% of HEU and 95.8% of HUU children. There were no significant differences in stunting between HEU and HUU children (23.5% vs. 23.0%, p=0.91). Neurodevelopmental assessments revealed no significant differences in gross motor, fine motor, language, or social domains. However, HEU carers had significantly lower education and income levels (p< 0.001). Low birth weight and prematurity were not associated with neurodevelopmental delays, irrespective of HIV exposure.

**Conclusion:**

HEU children did not show inferior nutritional or neurodevelopmental outcomes compared to HUU peers. Socioeconomic factors were more predictive of adverse outcomes. These findings highlight the need for integrated early childhood support systems addressing broader determinants of health beyond HIV exposure. In addition, strengthening community-based support systems and policies that empower women through education and employment could further enhance child health outcomes in resource-limited settings.

**Disclosures:**

All Authors: No reported disclosures

